# The Lateral Port Control Pharyngeal Flap: A Thirty-Year Evolution and Followup

**DOI:** 10.1155/2013/237308

**Published:** 2013-01-13

**Authors:** Sean Boutros, Court Cutting

**Affiliations:** ^1^Hermann Memorial Hospital and Hermann Children's Hospital, Houston, TX 77030, USA; ^2^Houston Plastic and Craniofacial Surgery, 6400 Fannin Suite 2290, Houston, TX 77030, USA; ^3^Institute for Reconstructive Plastic Surgery, New York University, New York, NY 10016, USA

## Abstract

In 1971, Micheal Hogan introduced the Lateral Port Control Pharyngeal Flap (LPCPF) which obtained good results with elimination of VPI. However, there was a high incidence of hyponasality and OSA. We hypothesized that preoperative assessment with videofluoroscopy and nasal endoscopy would enable modification and customization of the LPCPF and result in improvement in the result in both hyponasality and obstructive apnea while still maintaining results in VPI. Thirty consecutive patients underwent customized LPCPF. All patients had preoperative diagnosis of VPI resulting from cleft palate. Patient underwent either videofluoroscopy or nasal endoscopy prior to the planning of surgery. Based on preoperative velar and pharyngeal movement, patients were assigned to wide, medium, or narrow port designs. Patients with significant lateral motion were given wide ports while patients with minimal movement were given narrow ports. There was a 96.66% success rate in the treatment of VPI with one patient with persistent VPI (3.33%). Six patients had mild hyponasality (20 %). Two patients had initial OSA (6.67%), one of which had OSA which lasted longer than six months (3.33%). The modifications of the original flap description have allowed for success in treatment of VPI along with an acceptably low rate of hyponasality and OSA.

## 1. Introduction

In 1971, Micheal Hogan introduced the lateral port control pharyngeal flap [1–3]. This flap was conceived out of frustration over the inconsistent results obtained in the correction of velopharyngeal insufficiency with pharyngeal flaps. By noting important contributions to the understanding of physiology and dynamics of hypernasal speech by Warren, Isshiki, and Bjork [4–7], he devised a technique that could be universally applied to all patients with velopharyngeal insufficiency and obtain good result with consistent elimination of hypernasal speech [1–3, 8]. In his technique, the superiorly based flap, lined by the nasal side of the soft palate [9–12], was designed so that the lateral aperture size was controlled by the passage of a 4 mm diameter catheter. This effectively created an air passage that allowed the oropharyngeal pressure build up necessary to eliminate hypernasal speech.

After his initial description, the procedure evolved due to observation of the results. At the time Hogan described the LPC pharyngeal flap, sleep apnea had not yet been described as a clinical entity [13, 14]. In terms of speech intelligibility, hyponasality is preferred over hypernasality. The idea that many cleft palate patients with VPI often had good lateral wall movement allowing a “tailored width” pharyngeal flap [15] was also not yet widely known. For this reason, Hogan initially described a single-size flap that tended to produce very small lateral ports. Dr. Hogan intuitively began constructing larger ports in most patients and still maintained adequate results. In the past 30 years, the Hogan LPC flap became well known for the production of hyponasality and sleep apnea. The subsequent modifications of Hogan's original description, which takes these factors into account, are the subject of this paper. 

## 2. Materials and Methods

Thirty consecutive patients undergoing pharyngeal flap procedures for velopharyngeal insufficiency (VPI) were identified. Twenty-seven of these patients had VPI as a result of cleft palate, and 23 of these patients had adequate followup (greater than one year) for inclusion in this study. Patients were treated at the Institute of Reconstructive Plastic Surgery and either operated on or supervised by the senior surgeon (CC). Patients were treated according to the cleft VPI protocol as outlined later on. All patients were followed by the senior surgeon, pediatric otolaryngologist, and speech therapist.

### 2.1. Preoperative Evaluation

Patients with velopharyngeal insufficiency underwent evaluation with videofluoroscopy or fiberoptic nasal endoscopy [16, 17]. The findings are reviewed in a multidisciplinary clinic with a plastic surgeon, a pediatric otolaryngologist, and a speech therapist. Together, a consensus was reached as to the amount of velar and pharyngeal movement. 

In patients between 2.5 and 3 years of age who have not undergone intravelar veloplasty with the initial palate repair (palate closure performed at another institution), this procedure is the first-line treatment [18]. Many patients will attain adequate velar closure and have complete elimination of hypernasality with this procedure alone. These patients were excluded from this study.

In patients over four years, in whom the time course is more pressed due to the difficulty in elimination of compensatory articulations acquired after prolonged time with nasal escape, the pharyngeal flap is the procedure of choice if the nasal endoscopy shows lateral wall movement with poor central closure [8, 15–17, 19]. In a small minority of patients (none in our sample group), there may be good central movement with poor lateral closure. These rare patients are treated with a sphincter procedure. In addition, patients who have had previous intravelar veloplasty are also candidates for pharyngeal flaps. Based on the fiberoptic and videofluoroscopic findings, these patients are assigned to a small, medium-small, medium, medium-large, and large ports sizes. This corresponds with wide, medium-wide, medium, medium-narrow, and narrow pharyngeal flaps [13, 15, 17, 20, 21].

### 2.2. Operation

Prior to prep and drape, the posterior pharyngeal wall and the soft palate are infiltrated with approximately 10 cc of 0.5% lidocaine with 1 : 200,000 epinephrine. The posterior pharynx should always be palpated prior to infiltration, as patients with undiagnosed velo-cardio-facial syndrome are likely to have medialization of the carotid arteries, and care must be taken to avoid their injury. A Dingman's mouth gag is placed with the smallest tongue gag that will adequately hold the tongue on the floor of the mouth. Placement of a larger gag will limit the ability to reach the posterior pharyngeal wall. The handle of the gag is hung on the Mayo stand edge fully open and protrudes the mandible for optimal access. 

**Figure 1:**
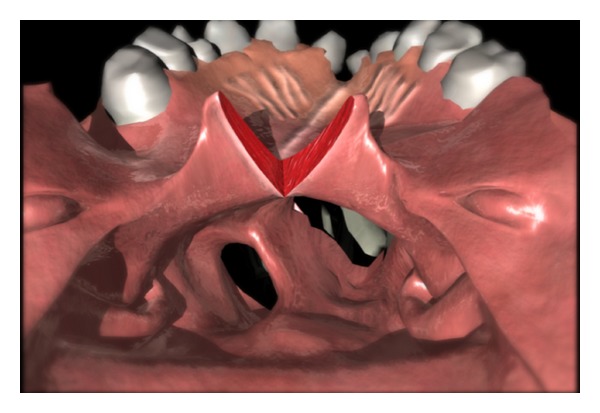
Division of soft palate.

The soft palate is split in the midline (Figure 1) and retraction sutures are placed. This split should stop just prior to the hard/soft palate junction (see supplementary video available online at doi:10.1155/2012/237308). The posterior pharyngeal wall is visualized, and the superiorly based pharyngeal flap of the appropriate width is outlined. As the flap is superiorly based, its mucosal surface will be reflected to the nasal side. It should be based as high as possible, approximately 15 mm caudal to the Eustachian tube orifices. The flap is incised to the parapharyngeal space. It is not necessary to incise to the prevertebral fascia as it does not contribute to the vascularity of the flap and results in a more painful donor site. The paired parapharyngeal spaces can be confirmed by the presence of the midline raphe. The flap is elevated with a peanut and the midline raphe cut with scissors. A suture is placed in the tip of the flap for retraction. 

The donor site should be closed directly except for the most proximal area. Closure decreases the postoperative pain, infection rate, and decreases downward migration of the flap with time. It will also allow for reestablishment of the sphincteric action of the pharyngeal wall with approximation of the muscles. It is best to close the middle of the donor site first and use the long end of a suture for retraction to expose the most caudal aspect of the donor site. Attempting to close the most cranial or proximal area of the donor site will cause the flap to take a tube shape and make creation of the appropriate size port difficult. Care must be taken to cauterize the edges of the cut posterior pharynx prior to closure as this is the most likely site of postoperative bleeding. This bleeding will most likely be from a cut ascending pharyngeal artery or one of its branches. Bleeding in this area may cause loss of airway and preclude oral intubation. Hemostasis is best performed with a suction cautery device. Although commercial suction cauteries are available, passing a neuro-tip suction through a red rubber catheter can easily create a suction cautery. 

**Figure 2:**
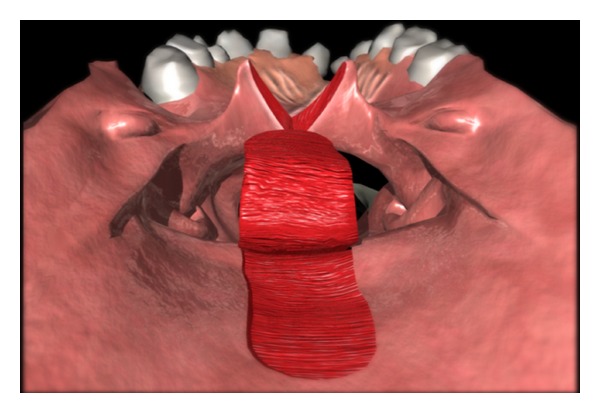
Elevation of superiorly based pharyngeal flap.

Attention should be turned to the creation of the lining flaps. It is important to consider the width of the lateral port much more than the width of the lining flap. The lining flaps are elevated from the nasal side of the soft palate and will line the oral side of the pharyngeal flap (Figure 2). They are based on the posterior edge of the soft palate, and the tip of the flap is at the hard/soft palate junction. The split soft palate should be reflected laterally. The rhomboidal-shaped flap is elevated off the underlying velar musculature. Starting at the most anterior aspect of the soft palate split, an incision is created toward the lateral/posterior edge of the soft palate. The lateral cut edge on the nasal surface of the soft palate will determine the size of the port. 

**Figure 3:**
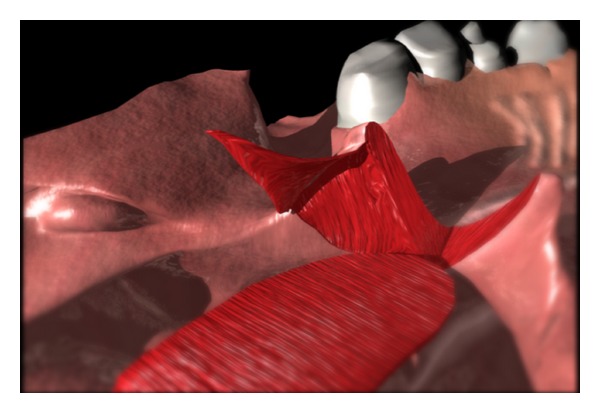
Elevation of the lining flap from the nasal side of the soft palate. Note that the lateral extent of the lining flap will help determine the size of the resulting lateral port.

**Figure 4:**
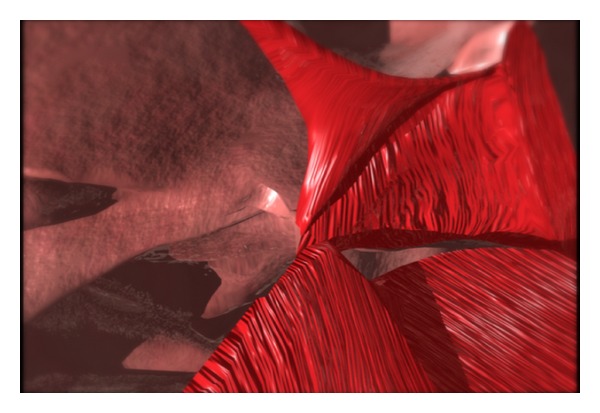
The key suture brings the lateral aspect of the lining flap to the superiorly based pharyngeal flap. This suture sets the size of the lateral port.

**Figure 5:**
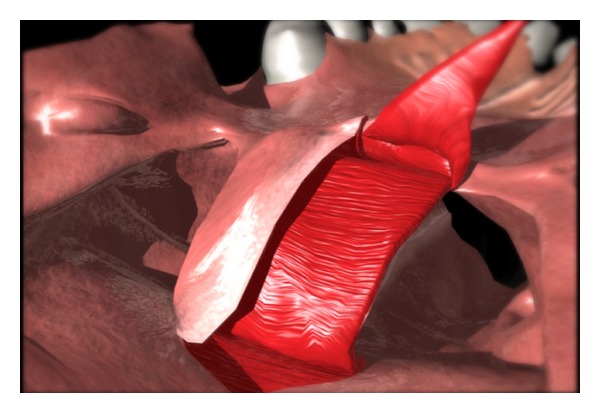
The lining flap is brought down to cover the raw side of the pharyngeal flap. This lining is crucial to prevention of contraction and tabularization of the pharyngeal flap.

The nasal lining flaps are then turned out to cover the raw surface of the pharyngeal flap. The port is created by suturing of the lateral cut edge to a point 5 mm from the base of the pharyngeal flap (Figures 3, 4, and 5). The lateral edge of the pharyngeal flap is sutured to the lateral cut edge from the elevation of the lining flaps, that is, the nasal side of the soft palate. The final suture is a horizontal mattress suture setting the tip of the flap well beyond the most anterior soft palate split in order to prevent formation of a fistula at this critical location. The suture is passed through and through (oral to nasal) the most anterior soft palate. It is passed through the tip of the pharyngeal flap in a mattress fashion and “through and through” (nasal to oral) the soft palate. 

The lateral edge of the tip of the lining flaps, elevated from the nasal side of the soft palate, is sutured to the lateral defect of the posterior pharyngeal wall. The medial edges of both lining flaps are sutured to the midline raphe at the base of the pharyngeal flap and to each other. The medial edges of the lining flaps are sutured together with each suture catching the midline raphe of the pharyngeal flap. The uvula is reconstructed, and the oral side of the soft palate is repaired.

A tongue stitch is placed in lieu of an oral airway as passage of an oral or nasal airway may disrupt the flap. The air and fluid are evacuated from the stomach, and blood is suctioned from the nose and pharynx. The patient is only extubated when fully awake, and the surgeon must be present in the room. After extubation, the patient is placed in a tonsillar position and kept awake. Traction on the tongue suture will both open the airway and stimulate the patient as needed.

### 2.3. Postoperative Management

In the initial postoperative period, airway observation is critical. The patients are kept on continuous pulse oximetry in the initial postoperative period. The intensive care unit is usually not required. The tongue suture is usually removed the next morning. Patients are given pain control with per rectum acetaminophen and codeine and kept on IV antibiotics to decrease the risk of streptococcal infection until they are taking liquids by mouth at which time they can be converted to oral antibiotics. They are allowed fluids immediately but are unlikely to take anything by mouth for the first few days. At the time they are taking adequate liquids, they can be discharged. The time course for oral intake varies dramatically. It ranges from three to nine days, but most patients take adequate fluids by mouth between three and four days. After several days of liquids, the patient is slowly transitioned to a soft diet, which is maintained for two to three weeks.

## 3. Results

Based on the preoperative evaluation of lateral wall motion, the procedures were divided as such: 6 patients had large port design (small flaps), 3 patients had large/medium port design (small/medium flaps), 14 patients had medium port design (medium flaps), and 6 patients had small/medium port design (medium/wide flaps). The incidence of small ports (wide flaps) was zero.

There was one patient with persistent VPI (4.3%). Five patients had mild hyponasality (21.7%). Two patients had initial sleep apnea (8.7%). One of the two had sleep apnea which lasted longer than six months (4.3%). This patient's flap was taken down with resolution of the VPI and no hypernasality. There was no airway compromise most likely due to hemostasis obtained prior to back wall closure. 

In all patients, there was some initial nocturnal obstruction due to swelling associated with the procedure. Overall, we have seen two patterns of sleep apnea in our patients. The first is obstruction at five to six weeks when wound contracture is at its highest. The obstruction resolves over several weeks as the contracture relaxes. There is a separate group in whom the contracture does not relax and there is resulting long-term obstruction. This may resolve over the next six to nine months, but if it does not resolve, the flap is taken down. Contraction of the pharyngeal flap may also lengthen the scarred soft palate [22].

## 4. Discussion

### 4.1. Preoperative Assessment

At the time of Hogan's original publication, there was no way to accurately assess the amount of velar or lateral pharyngeal movement preoperatively. The only measure of success was the postoperative result. As a result, in patients with some degree of pharyngeal movement, the results were typically good, and in patients with poor movement, the results were poor. There was no way to preoperatively stratify patients into the good or poor responder groups.

Videofluoroscopy and nasal endoscopy opened a new understanding of the movement of the velum and how surgical procedures could benefit patients [8, 16, 17, 23]. Videofluoroscopy allowed for direct visualization of the lateral pharyngeal wall movement, identifying the location and degree of the pathology and allowing formation of a reconstructive plan. This, along with the fundamentals of lateral port control technique of described by Hogan, allow for surgeons to customize the procedure to allow for appropriately sized flaps for each patient based on the amount of movement they have prior to surgery. This results in nasal competence, good speech, and limited hyponasality.

### 4.2. Port Diameter

Dr. Hogan was inspired to develop the lateral port control pharyngeal flap by the works of Drs. Warren and Isshiki. Both showed that the critical closing diameter allowing normal speech was 20 mm^2^ (Dr. Isshiki's critical diameter was 19.6 mm^2^). Dr. Hogan observed these facts and made two ports that would have a sum total of 25 mm^2^, (“…slightly larger than our threshold value of 20 mm^2^. Because of the mesial movement of the lateral pharyngeal walls which occurs during speech”) [19, 24]. In his design, Dr. Hogan focused on cross-sectional area of the ports not the airflow through the ports which is more important. According to Poiseuille's law, airflow is directly proportional to the fourth power of the radius. Thus, small changes in diameter have a dramatic effect on airflow.

(1) $$\begin{array}{c} \text{Flow}\;=\;\frac{\Pi \;\left(\text{pressure}\;\text{difference}\right)\;{\text{radius}}^{4}}{8\;\left(\text{viscosity}\right)\left(\text{length}\right)}. \end{array}$$

A 20 mm port has a radius of 2.526 mm,

(2) $$\begin{array}{c} \text{Flow}\;=\;\frac{\Pi \;\left(\text{pressure}\;\text{difference}\right){\left(2.526\;\text{mm}\right)}^{4}}{8\;\left(\text{viscosity}\right)\left(\text{length}\right)}. \end{array}$$

By this, half the flow or flow prime would be described by the following equation:

(3) Flow  prime=Flow2=Π(pressure  difference)(2.526 mm)416(viscosity)(length),Flow  prime  =Π(pressure  difference)(2.526 mm)416  (viscosity)(length),Π  (pressure  difference)(radius  prime)48  (viscosity)(length)  =Π  (pressure  difference)(2.526 mm)416(viscosity)(length),(radius  prime)48=(2.526)416,(radius  prime)4=20.36 mm,radius  prime  =  2.125 mm.

Therefore, two ports with a radius of 2.125 mm each (cross sectional area of 14.19 mm^2^ each and a total cross sectional area of 28.38 mm^2^) would have the same airflow as one port with a radius of 2.5 mm (cross sectional area of 19.63 mm^2^ each). 

In essence, a 50% larger sum total cross sectional area of two ports would have the exact same air flow resistance as a single port of 20 mm^2^. This of course assumes that there would be no pharyngeal movement. It can then be extrapolated that after pharyngeal flap, with the creation of ports, where with pharyngeal and velar movement the size of each of the two ports is reduced to an area of 14.19 mm^2^, there would be no clinically apparent hypernasality. 

In today's evaluation of the patient with velopharyngeal insufficiency, this becomes more significant as presurgical evaluation of the patient can give a much clearer picture of the pharyngeal movement. The procedure is no longer forced to address the least common denominator, that is, paralytic velum and pharyngeal wall, as it can be customized for each patient depending on the specific needs and level of dysfunction. 

Presently, the goal is to have complete velar closure. However, with two ports, even if there is not complete closure, the resulting nasal air escape would be less than that found with one port. 

### 4.3. Sleep Apnea

Not until recently did obstructive sleep apnea come into the attention of physicians treating velopharyngeal insufficiency [3]. Prior to this, nighttime obstruction and the resulting clinical symptoms after pharyngeal flap surgery were largely ignored, and the procedures touted as a success or failure solely on the effect on hypernasality. Nighttime snoring was even considered a measure of success as it indicated a low likelihood of nasal escape. However, more recent studies have shown that this important clinical entity is not only a source of significant morbidities including snoring, excessive daytime sleepiness, learning disabilities, irritability, perioperative aspiration pneumonia, growth retardation, heart disease, and hypertension, but also mortality with perioperative respiratory arrest and sudden death. 

The incidence of obstructive sleep apnea is controversial. Some authors report that with objective testing in a series of patients, over 90% will have some degree of sleep apnea after pharyngeal flap, and only some of which are clinically significant [25]. Most generally, it is quoted that an approximately 10% incidence of clinically apparent obstructive apnea and that only a fraction of these cases will require intervention [26–28]. In any event, the risk of postsurgical sleep apnea should be taken into account when approaching patients. With preoperative assessment and procedure individualization as we have shown, the incidence of clinically important sleep apnea can be significantly decreased resulting in few patients with this complication.

## 5. Conclusions

With some modifications from the original description by incorporation of preoperative diagnostic testing, the lateral port control pharyngeal flap has stood the test of time and has proven to be a powerful procedure in treatment of velopharyngeal insufficiency. Like all pharyngeal flaps, it serves to limit airflow from the oropharynx to the nasopharynx by forming an obstruction in the dysfunctional central area. It does not add any scarring or injury to the area where there is normal anatomy and the muscle function is good, that is, laterally. It uses this lateral pharyngeal sphincteric motion, along with the motion of the levator veli palatini muscle, to create a functional, dynamic obstruction to air flow. The lateral port control method turns the attention of the procedure to what is necessary for cure as it forces the surgeon to design a flap where the goal is the creation of a port of appropriate size that will prevent hypernasality while still resulting in an acceptable incidence of hyponasality and obstructive sleep apnea. 

## Supplementary Material

Video show animation and step by step illustration of the lateral port control pharyngeal flap.

## References

[1] Hogan VM (1973). A clarification of the surgical goals in cleft palate speech and the introduction of the Lateral Port Control (L.P.C.) pharyngeal flap. *Cleft Palate Journal*.

[2] Levine PA, Goode RL (1982). The lateral port control pharyngeal flap: a versatile approach to velopharyngeal insufficiency. *Otolaryngology*.

[3] Canady JW, Cable BB, Karnell MP, Karnell LH (2003). Pharyngeal flap surgery: protocols, complications, and outcomes at the University of Iowa. *Otolaryngology*.

[4] Warren DW (1964). Velopharangeal orifice size and upper pharangeal pressure flow patterns in normal speech. *Plastic and Reconstructive Surgery*.

[5] Warren DW, Devereux JL (1966). An analog study of cleft palate speech. *Cleft Palate Journal*.

[6] Isshiki N, Honjow I, Morimoto M (1968). Effects of velopharyngeal incompetence upon speech. *Cleft Palate Journal*.

[7] Bjork L Velopharangeal function in connected speech.

[8] Cable BB, Canady JW (2003). The endoscopically assisted pharyngeal flap. *Cleft Palate-Craniofacial Journal*.

[9] Stoll C, Hochmuth M, Meister P, Soost F (2000). Refinement of velopharyngoplasty in patients with cleft palate by covering the pharyngeal flap with nasal mucosa from the velum. *Journal of Cranio-Maxillofacial Surgery*.

[10] Newman FJ, Messinger A (1985). A method for lining the superiorly based pharyngeal flap. *Annals of Plastic Surgery*.

[11] Vandevoort MJ, Mercer NS, Albery EH (2001). Superiorly based flap pharyngoplasty: the degree of postoperative ’tubing’ and its effect on speech. *British Journal of Plastic Surgery*.

[12] Hardy SB, Spira M (1983). Lining the superiorly based pharyngeal flap. *Annals of Plastic Surgery*.

[13] Daly DD, Yoss RE (1965). Pathologic sleep. *International Journal of Neurology*.

[14] Brouillette RT, Fernbach SK, Hunt CE (1982). Obstructive sleep apnea in infants and children. *Journal of Pediatrics*.

[15] Argamaso RV, Shprintzen RJ, Strauch B (1980). The role of lateral pharyngeal wall movement in pharyngeal flap surgery. *Plastic and Reconstructive Surgery*.

[16] Crockett DM, Bumsted RM, Van Demark DR (1988). Experience with surgical management of velopharyngeal incompetence. *Otolaryngology*.

[17] Sommerlad BC, Mehendale FV, Birch MJ, Sell D, Hatte C, Harland K (2002). Palate re-repair revisited. *Cleft Palate-Craniofacial Journal*.

[18] Shprintzen RJ, Lewin ML, Croft CB (1979). A comprehensive study of pharyngeal flap surgery: tailor made flaps. *Cleft Palate Journal*.

[19] Karnell MP, Ibuki K, Morris HL, Van Demark DR (1983). Reliability of the nasopharyngeal fiberscope (NPF) for assessing velopharyngeal function: analysis by judgment. *Cleft Palate Journal*.

[20] Peat BG, Albery EH, Jones K, Pigott RW (1994). Tailoring velopharyngeal surgery: the influence of etiology and type of operation. *Plastic and Reconstructive Surgery*.

[21] Shprintzen RJ, McCall GN, Skolnick ML (1980). The effect of pharyngeal flap surgery on the movements of the lateral pharyngeal walls. *Plastic and Reconstructive Surgery*.

[22] Agarwal T, Sloan GM, Zajac D, Uhrich KS, Meadows W, Lewchalermwong JA (2003). Speech benefits of posterior pharyngeal flap are preserved after surgical flap division for obstructive sleep apnea: experience with division of 12 flaps. *The Journal of Craniofacial Surgery*.

[23] Ysunza A, Pamplona MC, Ramírez E, Canún S, Sierra MC, Silva-Rojas A (2003). Videonasopharyngoscopy in patients with 22q11.2 deletion syndrome (Shprintzen syndrome). *International Journal of Pediatric Otorhinolaryngology*.

[24] Karling J, Henningsson G, Larson O, Isberg A (1999). Adaptation of pharyngeal wall adduction after pharyngeal flap surgery. *Cleft Palate-Craniofacial Journal*.

[25] Lia YF, Chuang ML, Chen PK, Chen NH, Yun C, Huang CS (2002). Incidence and severity of obstructive sleep apnea following pharyngeal flap surgery in patients with cleft palate. *Cleft Palate-Craniofacial Journal*.

[26] Wells MD, Vu TA, Luce EA (1999). Incidence and sequelae of nocturnal respiratory obstruction following posterior pharyngeal flap operation. *Annals of Plastic Surgery*.

[27] Sirois M, Caouette-Laberge L, Spier S, Larocque Y, Egerszegi EP (1994). Sleep apnea following a pharyngeal flap: a feared complication. *Plastic and Reconstructive Surgery*.

[28] Ysunza A, Garcia-Velasco M, Garcia-Garcia M, Haro R, Valencia M (1993). Obstructive sleep apnea secondary to surgery for velopharyngeal insufficiency. *Cleft Palate-Craniofacial Journal*.

